# Bioactive Natural Matrices and Compounds

**DOI:** 10.1155/2014/125964

**Published:** 2014-02-03

**Authors:** Isabel C. F. R. Ferreira, Marina Soković, Lillian Barros

**Affiliations:** ^1^Mountain Research Center (CIMO), ESA, Polytechnic Institute of Bragança, Campus de Santa Apolónia, Apartado 1172, 5301-855 Bragança, Portugal; ^2^Department of Plant Physiology, Institute for Biological Research “Siniša Stanković”, University of Belgrade, Bulevar Despota Stefana 142, 11000 Belgrade, Serbia

The demand for healthy ingredients and a natural way of preventing diseases is contributing to the increased use of natural matrices. There has been a lot of research indicating that general antioxidant properties are not the contributing factors to improving human health but rather the antioxidant properties of specific compounds involved in specific cell signaling mechanisms that need to be elucidated. In this way, beyond the composition of the usual macronutrients and micronutrients, it seems important to also provide information on the composition of natural bioactive compounds. Phytochemicals such as phenolic compounds, essential oils, steroids, vitamins, or pigments should assume high importance due to their documented antioxidant, antimicrobial, antitumour, and anti-inflammatory properties, among others, but the knowledge of the mechanisms of action involved is mandatory. The contributions for this special issue consist of five papers covering several aspects of nutraceuticals and phytochemicals in natural matrices with proven activities in various *in vitro* or *in vivo* models.

The paper by R. P. Constantin et al. evaluated the effects of three citrus flavanones (hesperidin, hesperetin, and naringenin) on several parameters linked to fatty acid oxidation in mitochondria, peroxisomes, and perfused livers of rats. These results confirm the hypothesis that citrus flavanones are able to induce a more oxidised state in liver cells, altering parameters related to hepatic fatty acid oxidation. The prooxidant effect is most likely a consequence of the ability of these substances to oxidise NADH upon production of phenoxyl radicals in the presence of peroxidases and hydrogen peroxide.

A. Chennuru and M. T. S. Saleem described the cardioprotective effects of sesamol against doxorubicin-induced cardiomyopathy in rats. The authors concluded that, sesamol has significant cardioprotection against doxorubicin that induced cardiomyopathy via amelioration of oxidative stress, lipid lowering, and membrane stabilization effect.

C. Chaotham et al. evaluated the oral toxicity of a partially purified plaunotol extract (PPE), which is traditionally consumed for its antigastric ulcer properties, using *in vivo* assays. The acute toxicity study demonstrated that PPE was practically nontoxic based on its high median lethal dose value. The chronic toxicity studies also showed the absence of mortality and clinical symptoms in all treated rats. Therefore, the authors concluded that these results suggest that PPE is potentially safe for further development as a therapeutic agent in humans.

In the research study presented by H. A. E. Rabey et al., the protective effect of natural bees' honey on the liver of male albino rats against melamine toxicity was reported. Treating the male albino rats (that were presupplemented regularly with 20000 ppm melamine) with natural bees' honey at a dose of 2.5 g/kg body weight for 28 days improved both liver functions and increased serum protein. In addition, a positive impact on the shape of the cells after treatment with honey, compared to the positive melamine supplemented group, was observed. The authors concluded that the use of natural bees' honey has the ability to protect the livers of rats against the toxic effects of melamine.

A. Rasul et al. reviewed recent data on primary flavonoids isolated from plants. Pinocembrin exhibits pharmacological effects on almost all systems, and the authors reviewed its pharmacological and therapeutic applications with specific emphasis on mechanisms of actions. The review suggested that pinocembrin is a potentially promising pharmacological candidate, but additional studies and clinical trials are required to determine the specific intracellular sites of action and derivative targets in order to fully understand the mechanism of its anti-inflammatory, anticancer, and apoptotic effects to further validate pinocembrin medical applications.

